# H atom scattering from W(110): A benchmark for molecular dynamics with electronic friction.†

**DOI:** 10.1039/d2cp01850k

**Published:** 2022-08-25

**Authors:** Raidel Martin-Barrios, Nils Hertl, Oihana Galparsoro, Alexander Kandratsenka, Alec M. Wodtke, Pascal Larrégaray

**Affiliations:** Univ. Bordeaux, CNRS, Bordeaux INP, ISM, UMR5255 F-33400 France pascal.larregaray@u-bordeaux.fr; Dynamical processes in Atomic and Molecular Systems (DynAMoS), Facultad de Física, Universidad de la Habana La Habana 10400 Cuba; Max-Planck Institut für multidisziplinäre Naturwissenschaften, Am Faßberg 11 Göttingen Germany nils.hertl@mpinat.mpg.de; Institut für physikalische Chemie, Georg-August-Universität, Tammannstraße 6 Göttingen Germany; Polimero eta Material Aurreratuak: Fisika, Kimika eta Teknologia Saila, Kimika Fakultatea, Euskal Herriko Unibertsitatea (UPV/EHU) Lardizabal Pasealekua 3 20018 Donostia-San Sebastián Spain

## Abstract

Molecular dynamics with electronic friction (MDEF) at the level of the local density friction approximation (LDFA) has been applied to describe electronically non-adiabatic energy transfer accompanying H atom collisions with many solid metal surfaces. When implemented with full dimensional potential energy and electron density functions, excellent agreement with experiment is found. Here, we compare the performance of a reduced dimensional MDEF approach involving a simplified description of H atom coupling to phonons to that of full dimensional MDEF calculations known to yield accurate results. Both approaches give remarkably similar results for H atom energy loss distributions with a 300 K W(110) surface. At low surface temperature differences are seen; but, quantities like average energy loss are still accurately reproduced. Both models predict similar conditions under which H atoms that have penetrated into the subsurface regions could be observed in scattering experiments.

## Abstract 2

Molecular dynamics with electronic friction (MDEF) at the level of the local density friction approximation (LDFA) has been applied to describe electronically non-adiabatic energy transfer accompanying H atom collisions with many solid metal surfaces.

## Graphical abstract

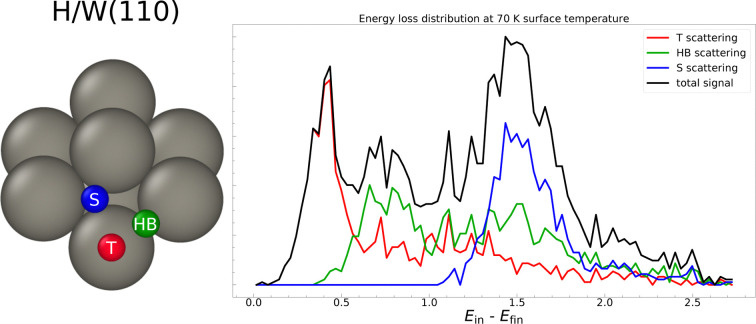

## 1 Introduction

The dynamics of elementary processes of atoms and molecules interacting at metal surfaces has been intensively studied in order to gain a deeper understanding of catalysis.^1,2^ H atom scattering on clean^3–8^ and covered^9–16^ transition metal surfaces exhibit non-adiabatic energy transfer between the incident atom's translational motion and electron–hole pair (ehp) excitation. In contrast to ehps, phonons dissipate energy less effectively because of the large mass difference between hydrogen and transition metal atoms. In molecular dynamics (MD) simulations, ehp excitation is often modelled by a frictional force exerted on the H atom moving classically on a single potential energy surface (PES). The friction coefficient is commonly calculated within the framework of the local density friction approximation (LDFA).^17–20^ Full dimensional simulations successfully reproduce energy loss and angular scattering distributions obtained in high resolution experiments for a variety of metals.^4^*Ab initio* molecular dynamics with electronic friction (AIMDEF)^21–23^ allows such calculations on-the-fly and in full-dimensions; however, such calculations can be computationally costly. Hence, for many problems, fast and reliable pre-computed potential energy surfaces (PESs) are used – high-dimensional neural networks now make this much easier than in the past.^24–27^ Nevertheless, even this approach is sometimes difficult – large amounts of density functional theory (DFT) data are needed, and the fitting procedure is time-consuming. It is therefore often useful to use simplified physical model potentials. Therefore, rigorous investigations of their reliability are necessary.

In this work, we compare the performance of two established model potentials which are based on different theoretical frameworks.^13,28^ Both model potentials have been proven to be suitable for modelling the electronically non-adiabatic scattering dynamics of atomic hydrogen at metal surfaces. To accomplish our aim, we test the potentials on the same observables, *i.e.* energy loss distributions (ELDs) and identify possible discrepancies. The first, effective medium theory (EMT)^29,30^ is a full dimensional PES approach previously used for H atom scattering from fcc(111) surfaces.^3,4,31^ It models excitation of phonons by full dimensional classical MD and the frictional force is computed from EMT-background electron densities associated with the moving lattice. The second is a corrugation reduction procedure (CRP),^32–34^ which has also been applied to H atom scattering from metal surfaces^7^ as well as hydrogen recombination.^9–16^ The CRP-PES approach treats electronic friction using a single electron density function for a frozen lattice computed with DFT. Excitation of phonons is approximated by a generalised Langevin oscillator (GLO) model.^35,36^ We find that the two used approaches give similar ELDs for H scattering from a W(110) surface. This shows that important experimental observables can be accurately predicted with a simplified reduced dimensional simulation method.

## 2 Methods

We implemented MDEF to simulate the full dimensional scattering of H from W(110) using the Langevin equation:1
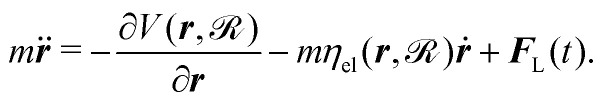
Here ***r*** and ℛ are the position of the H-atom and the positions of the metal atoms, respectively. *V* (***r***, ℛ) is the potential energy of the system, *m* is the mass of the projectile, *η*_el_(***r***, ℛ) is the electronic friction coefficient, and ***F***_**L**_(*t*) is the random force, which obeys the fluctuation-dissipation theorem.^37^ We computed the frictional force within the LDFA.^17–20^

For our EMT-based MDEF simulations, we constructed a full-dimensional PES as has been previously described^28^ using DFT data generated with the PBE functional^38,39^ and the projector-augmented wave (PAW) approach for electron-core interactions.^40^ A plane wave basis set with a cut-off energy of 400 eV was used. Partial occupancies were modelled with the method of Methfessel-Paxton^41^ (*N* = 1) and a smearing width of 0.1 eV was applied. The *k*-point grid for the Brillouin zone of W(110) has been sampled with a (12 × 12 × 1) Monkhorst-Pack mesh.^42^ DFT data used to fit the EMT PES included structures where the tungsten atoms were fixed at their equilibrium positions, as well as structures from several AIMD trajectories where all atoms were allowed to move. The fitted full-dimensional EMT function also provides the full-dimensional background electron density. The dependence of *η*_el_(***r***, ℛ) on the electron density was taken from ref. 5. The motion of the metal slab atoms is governed by Newton's equations using the full dimensional EMT potential. Coupling of the metal atom's motion to the electrons is neglected.

**Fig. 1:**
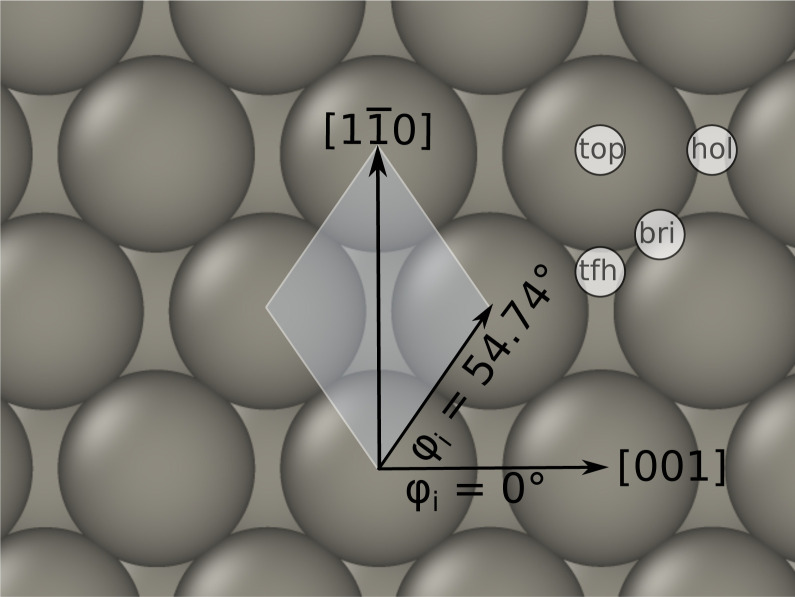
Surface geometry of W(110) associated with the coordinate system. The azimuthal angle is defined with respect to the [001] direction. Note that this direction corresponds to the *x*-axis of our simulation cells. The white shaded area marks the *p* (1 × 1) unit cell. Top (top), hollow (hol), bridge (bri), three fold hollow (tfh) high symmetry sites are also indicated.

Trajectories within the EMT model were initiated using structures and W atom velocities sampled from a thermal ensemble produced as follows. First, the slab was heated with an Anderson-thermostat^43^ for 100 ps and subsequently equilibrated in a microcanonical manner for another 100 ps, using the Verlet algorithm.^44,45^ Once thermalisation was confirmed, the trajectory was run for 1 ns picking structures every picosecond to produce an ensemble of 1000 structures. H atom's initial positions were chosen at *Z*_p_ = 6 Å above the surface and randomly in the *xy*-plane. Fig. 1 depicts the coordinate system used in this work.

The CRP-PES used here^13^ relies on the established interpolation scheme, which is described elsewhere,^32,46,47^ to mimic the interaction between an H atom with a rigid slab of W atoms at their equilibrium positions. The electron density for the bare, rigid W(110) slab is then used for the Langevin dynamics. DFT calculations were performed using the PW91 exchange-correlation functional^48,49^ and parameters detailed previously.^13,47^ Interactions with the atomic cores were described by ultrasoft pseudopotentials (US).^50^ A five-layer W slab for which the inter-layer distances have been optimised, was employed to represent a (2 × 2) cell with 15 Å vacuum space between consecutive slabs. The *k*-point sampling of the Brillouin zone made use of a Monkhorst-Pack (5 × 5 × 1) grid and an electron smearing of 0.4 eV was introduced. The influence of the metal's phonons was modelled by a generalised Langevin oscillator (GLO) which has been detailed elsewhere.^36,51–53^ A comparison of one-dimensional potential energy curves of both PESs can be found in the ESI.†

Trajectories within the CRP-PES approach are computed similarly as described above; however, here the position and momentum of the GLO are sampled from a Boltzmann distribution. The GLO is given an effective mass equal to that of a single W atom. The GLO is coupled to a thermal bath, which is composed of a ghost atom subject to friction and random forces.^36,51–53^ To ensure convergence, more than 10^6^ trajectories have been run.

Though the interpolation of DFT data is more accurate within the CRP-PES, the model neglects the explicit motion of W atoms^13,47^ as accounted in the EMT-PES.^28^ It is thus highly relevant to compare the prediction of both models on the same observables, as performed in the present work.

## 3 Results

**Fig. 2:**
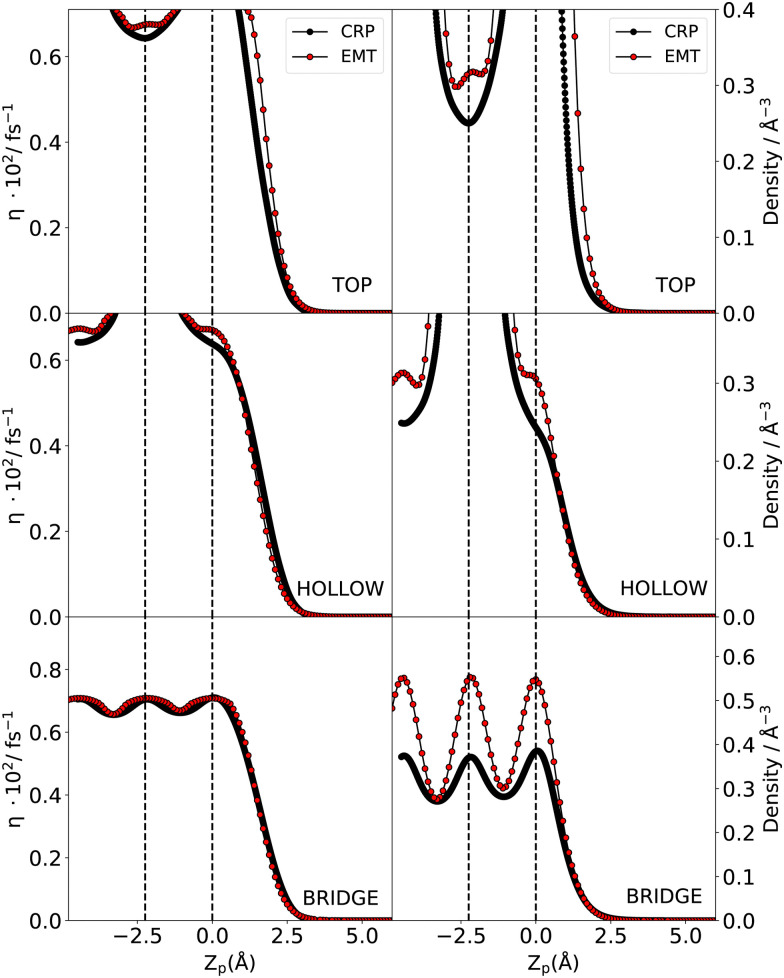
Comparison of the friction coefficient (left) and electron density (right) for the top, hollow and bridge high symmetry sites from CRP (black) and EMT (red) models. The high symmetry sites are depicted in Fig. 1. Note that Z_p_ = 0 corresponds to the location of the surface.

Fig. 2 compares the electron densities and friction coefficients for the two models where the H atom is brought along the normal to the surface over the top, bridge and hollow high symmetry sites. See also Fig. 1 for a visualisation of the surface structure. Both quantities are higher for the EMT model. Present experimental setups are designed to only detect energy loss distributions for in-plane scattered atoms.^54^ Through theory, no such restriction is necessary; hence, we also present angle integrated energy loss distributions in this work.

### 3.1 Angle-averaged energy loss distributions

**Fig. 3:**
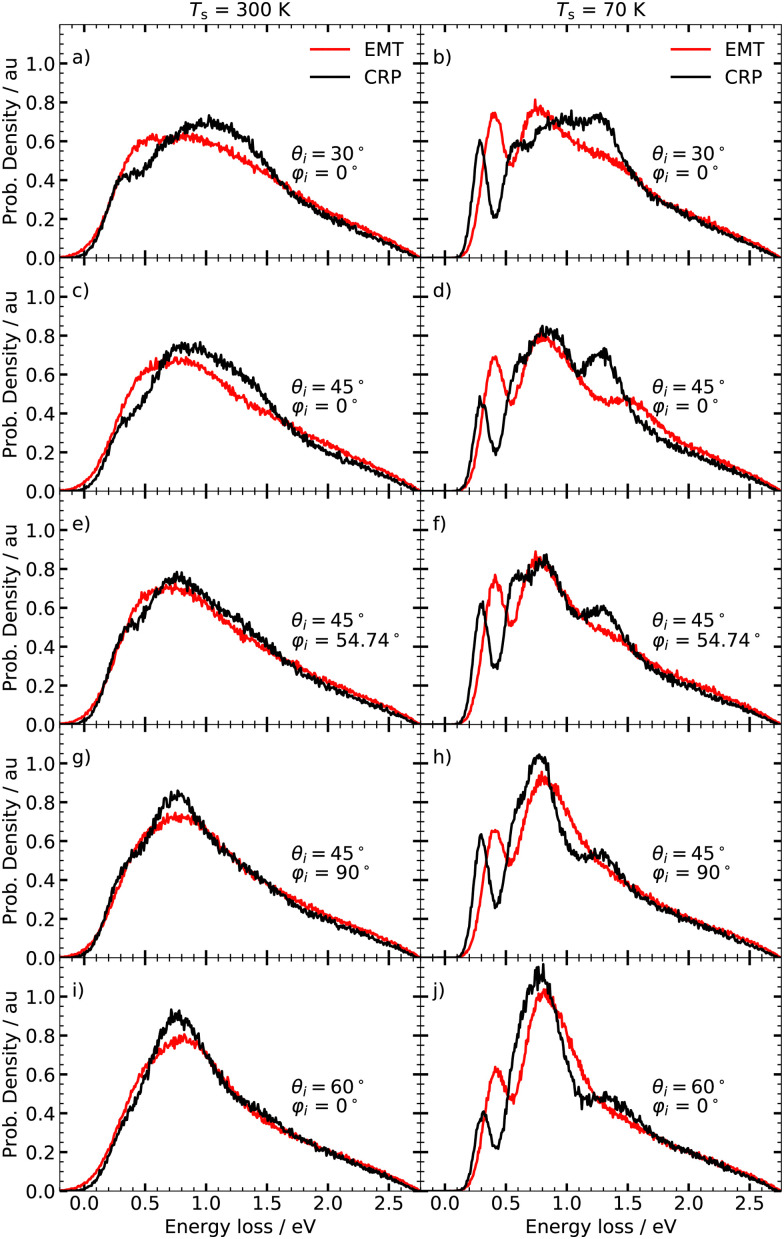
Angle integrated energy loss distribution at *T*_s_ = 300 K (left panel) and 70 K (right panel) for various polar angles *θ*_i_ and azimuthal angles *φ*_i_. *φ*_i_ = 0° corresponds to the [001] direction. The collision energy *E*_c_ is 2.76 eV.

Fig. 3 displays the angle integrated ELDs obtained under various incidence conditions at two surface temperatures; room temperature (left panels) and 70 K (right panels). We do not take thermal expansion of the lattice constant for tungsten into account as this effect is reported to be weak at ambient temperatures.^55,56^ At room temperature, both models yield broad, structureless ELDs that are in good agreement with each other. Pronounced differences between the two models can be seen at *T*_s_ = 70 K. Although they both yield distributions comprising characteristic peaks, the EMT-based calculations predict that the individual peaks are shifted to higher energy losses. The fine structure of the ELDs depends further on the initial polar angle *θ*_i_ and the initial azimuthal angle *φ*_i_. The ELDs calculated with the CRP-PES are more sensitive to the initial conditions compared to their EMT counterparts. In particular, the intensity of the peak at energy losses between 1.25 and 1.5 eV shows a pronounced dependence on *φ*_i_ – see panels d), f) and h) of Fig. 3.

**Fig. 4:**
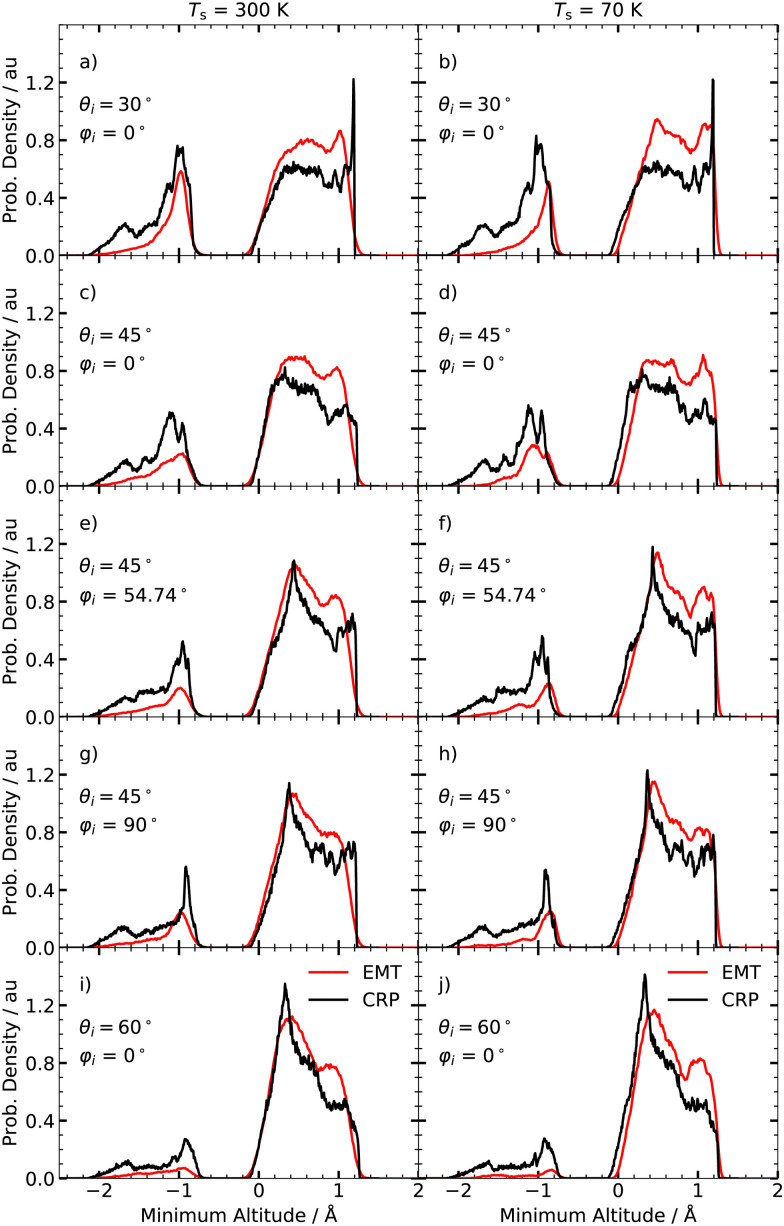
Angle averaged minimum altitude distributions at *T*_s_ = 300 K (left panel) and 70 K (right panel) for various polar angles *θ*_i_ and azimuthal angles *φ*_i_. The red and black curves show the results obtained with the EMT and CRP-model, respectively. Note that *φ*_i_ = 0° corresponds to the [001] direction and *Z*_min_ = 0 Å is the location of the surface. The collision energy *E*_c_ is 2.76 eV. The distributions are normalised with respect to the area under the curves.

Fig. 4 shows the minimum altitude distributions acquired from the simulations used for the calculation of the ELDs shown in Fig. 3. Both models predict that the majority of scattered H atoms bounce back from the surface layer. Still, a significant portion of projectiles underwent surface penetration. This corresponds to negative *Z*_min_ values in Fig. 4. The fraction of penetrated particles *F*_p_ increases with decreasing polar angle *θ*_i_, because of the increased normal kinetic energy *E*_c_cos ^2^*θ*_i_. In contrast to the ELDs, the shape of minimum altitude distributions is barely affected by the surface temperature. It is worth noting that the CRP model predicts a higher fraction of H atoms that penetrate the surface.

### 3.2 Subsurface penetration and sticking

**Fig. 5:**
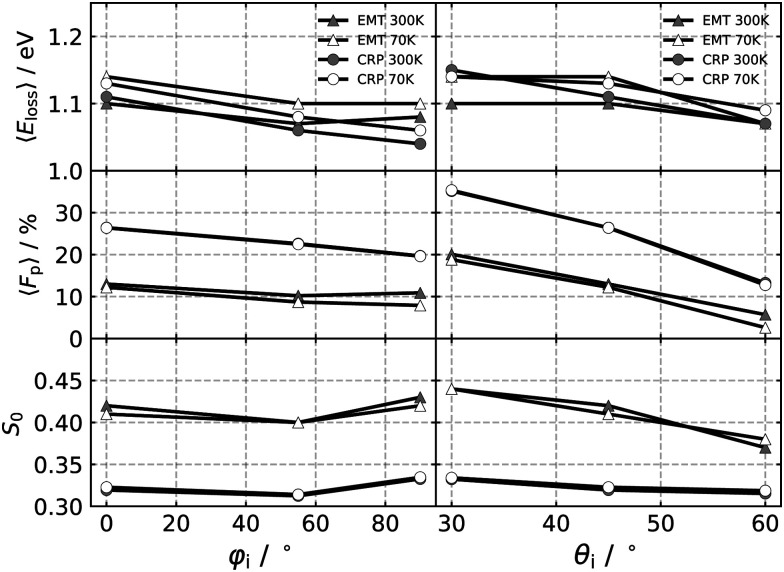
Mean energy loss 〈*E*_loss_〉, fraction of scattered particles after penetration into the surface *F*_p_ and sticking coefficient *S*_0_ as a function *φ*_i_ with *θ*_i_ = 45° (left) for *E*_c_ = 2.76 eV. Same quantities as a function of *θ*_i_ with *φ*_i_ = 0° (right). 〈*E*_loss_〉 and *F*_p_ were calculated from all scattering events.

The average energy loss 〈*E*_loss_〉, the fraction of atoms scattered after penetration into the surface *F*_p_, and the sticking coefficient *S*_0_, are displayed in Fig. 5 as a function of the azimuthal and polar angles for 70 K and 300 K surface temperatures. Both models predict similar 〈*E*_loss_〉 values, which are slightly higher at low temperature, due to the reduced influence of the random force.^8^ The influence of changing *θ*_i_ and *φ*_i_ is weak. For all initial conditions, the energy transfer to ehps is the dominant energy transfer channel. However, the energy transfer to the phonons differ between both models. In the CRP-GLO framework, phonon excitations make up only 3% of the mean energy loss. Explicit treatment of the metal atoms allowed by the EMT-PES gives rise to larger energy losses to the lattice. On average 16% of the mean energy loss is taken up by the lattice, but therefore the mean energy loss to the electrons is somewhat smaller compared to the CRP model – see Fig. 3 in the ESI.† This compensation effect between both energy transfer channels helps explain why both models predict similar mean energy losses.

Simulations performed with CRP-PES show generally a larger probability for subsurface scattering events, represented by the fraction penetrated particles, *F*_p_, for all investigated initial conditions compared to their EMT analogues. Both models predict similar trends for *F*_p_; the fraction of surface penetration decreases with increasing *φ*_i_ and *θ*_i_. Moreover, this trend is temperature independent for the CRP-PES method, but weakly temperature dependent for the EMT-PES method.

The EMT-PES method predicts higher *S*_0_ values compared to the CRP-PES. Both models predict that *S*_0_ is barely affected by changes to *φ*_i_. Simulations with EMT-PES predict smaller *S*_0_ values for increasing *θ*_i_, consistent with previous simulations.^4^

### 3.3 Specular scattering dynamics

**Fig. 6:**
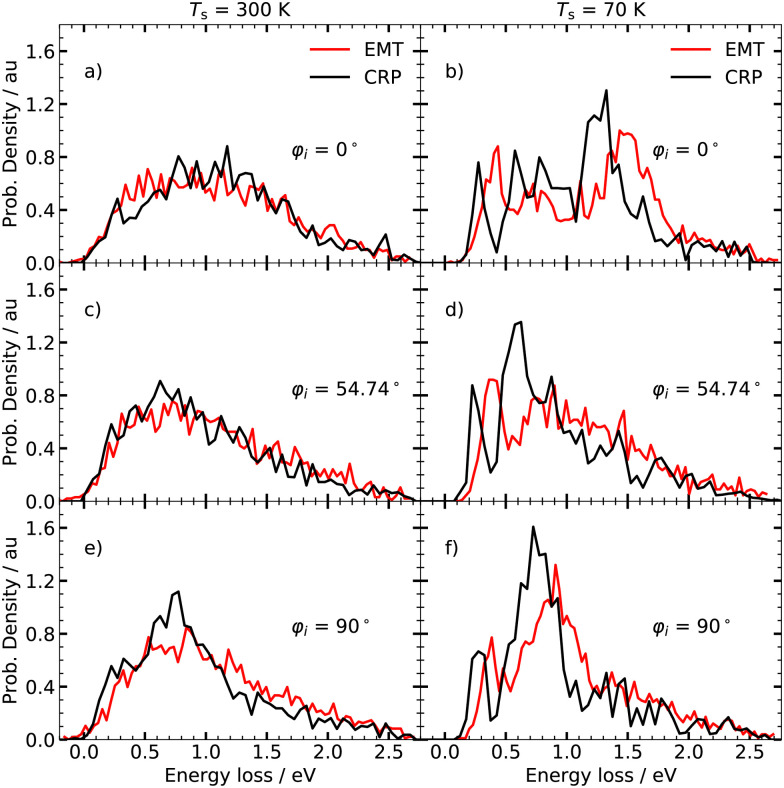
Specular ELD for the CRP (black) and EMT (red) models respectively at *T*_s_ = 300 K (left panels) and *T*_s_ = 70 K (right panels). The polar angle of incidence is *θ*_i_ = 45° and collision energy is *E*_c_ = 2.76 eV.

To motivate possible future experiments, in this section we present predicted ELDs under experimentally realisable conditions.^4,54^ We extract particle trajectories scattered to defined polar *θ*_f_ and azimuthal *φ*_f_ angles – for simplicity, we consider only experiments where *θ*_f_ = *θ*_i_ = 45° and *φ*_f_ = *φ*_i_. We accept trajectories that will strike a detector with an acceptance of angle of ±5°. The ELDs for *T*_s_ = 300 K and 70 K are shown in Fig. 6.

The specular ELDs obtained using the EMT-PES at *T*_s_ = 300 K are broad and structureless, resembling those for H atom scattering from the fcc metals.^4^ The CRP-based model yields similar looking specular ELDs, but the results are more sensitive to the choice of *φ*_i_ compared to the EMT-based model. At *T*_s_ = 70 K, clear differences appear between the specular and angle-integrated ELDs depicted in Fig. 3. At *φ*_i_ = 0°, three features are again seen, but for the specular ELD, the peak at ∼ 1.5 eV is enhanced, whereas it vanishes for *φ*_i_ = 54.74° and *φ*_i_ = 90°. Although both models predict specular ELDs of similar shape, the distributions differ in intensity and location of the individual peaks – see right panels of Fig. 6.

## 4 Discussion

**Fig. 7:**
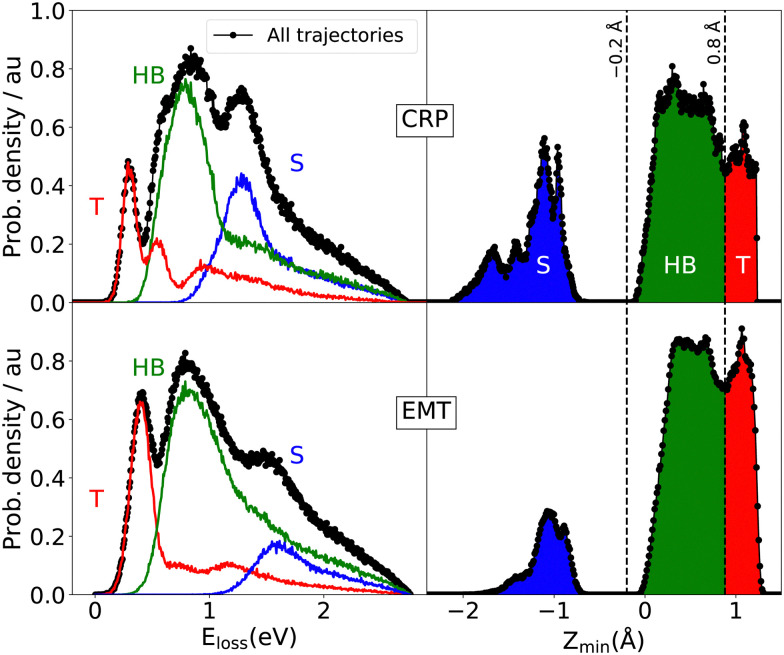
Angle integrated energy loss distributions (ELD, left panels) and minimum turning point altitude distribution (AD, right panels) for all scattered atoms for the CRP (top) and EMT (down) models. Red, green and blue colours respectively correspond to the T(Top), HB(Hollow and Bridge) and S(Subsurface) contributions. These distributions correspond to *T*_s_ = 70 K and *E*_c_ = 2.76 eV collision energy at *θ*_i_ = 45° and *φ*_i_ = 0°.

We first consider the peak structure for the predicted ELDs at low *T*_s_ seen in Fig. 3. Analysing trajectories reveals a strong correlations between *E*_loss_ and *Z*_min_ – see Fig. 7. T scattering takes place on top of tungsten surface atoms exhibiting values of *Z*_min_ > 0.8 Å. HB reflects trajectories scattering from hollow and bridge sites, where −0.2 Å < *Z*_min_ < 0.8 Å. S scattering denotes trajectories that pass through the subsurface regions (−2.1 Å < *Z*_min_ < −0.2 Å) and bounce off second layer tungsten atoms lying below the four-fold hollow symmetry surface sites at *Z*_min_ = −2.24 Å. The contributions of T, HB and S scattering to the angle-integrated ELDs are shown in the left panel of Fig. 7. T scattering leads to the smallest energy losses. HB scattering involves a longer interaction time with more efficient excitation of ehps. S scattering gives by far the largest energy losses (>1 eV).

**Fig. 8:**
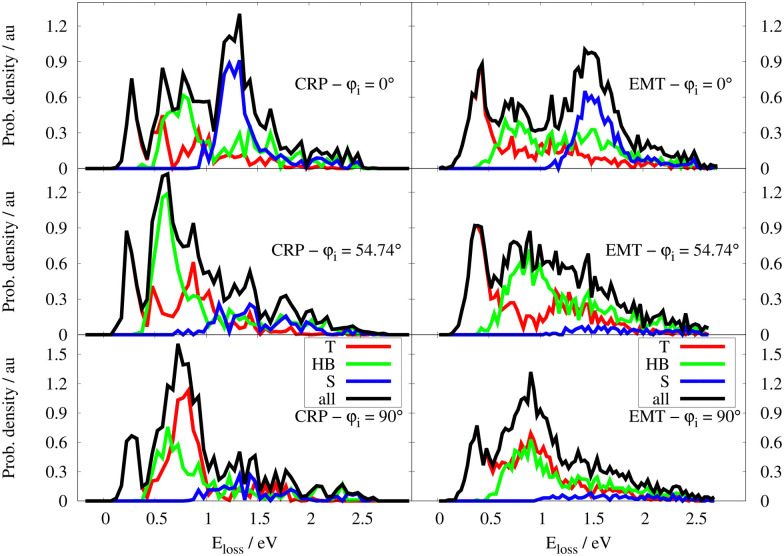
Specular ELD for the CRP (left) and EMT (right) models respectively at *T*_s_ = 70 K for *φ*_i_ = 0°,54.74° and 90°. ELDs are decomposed into the T, HB, S contributions (see text). The collision energy is *E*_c_ = 2.76 eV and *θ*_i_ = 45°.

Similar analysis was performed for the specular ELDs at 70 K – see Fig. 8. Again, the peak exhibiting the lowest energy loss results from a central collision between projectile and surface atoms for all initial incidence directions. Both scattering models also predict that the subsurface channel results in a distinct peak at 1.5 eV energy loss and that this feature is sensitive to *φ*_i_. An incidence direction corresponding *φ*_i_ = 0° favours subsurface scattering. If the H atoms approach the W surface along the closed-packed direction, *i.e. φ*_i_ = 54.74°, the amount of specular subsurface scattering is drastically reduced. At *φ*_i_ = 90°, both models yield a smaller subsurface contribution to the signal compared to *φ*_i_ = 0° reflecting the critical orientation dependence of this subsurface scattering channel. The fraction of specular scattered H atoms which underwent surface penetration, denoted as *F*_p,spec_, is presented in Table 1. We emphasise that this is the first prediction that subsurface scattering might be detected in H scattering from a metal. Experimentally confirming this prediction would further validate the models presented in this work.

**Table 1:** Contribution of subsurface scattered H atoms to the specular energy loss distribution *F*_p,spec_ in (%) as a function of the initial azimuthal angle *φ*_i_ for both employed MDEF frameworks at 300 K (70 K). The other initial conditions are *θ*_i_ = 45° and *E*_c_ = 2.76 eV

*φ*_i_ (°)	0	54	90
EMT	17.6 (27.5)	7.26 (3.78)	8.44 (5.46)
CRP	32.4 (31.9)	18.7 (18.3)	15.5 (14.4)

Despite the qualitative agreement between both scattering models, quantitative differences in the ELDs are seen. The higher electron density of the EMT model shifts the peaks of the T, HB and S scattering channels towards higher energy losses. This also gives rise to an increased sticking probability and a lower contribution of subsurface scattering compared to the three-dimensional CRP model – see Fig. 5 and Table 1.

**Fig. 9:**
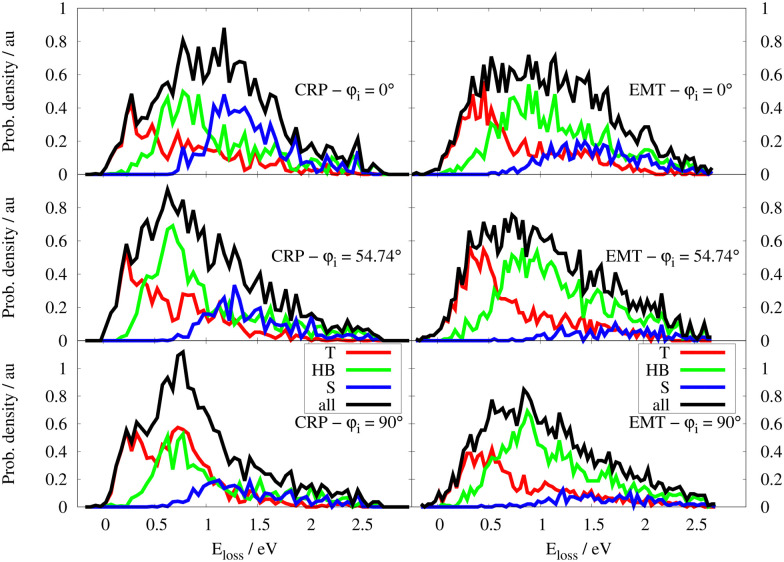
Specular ELD for the CRP (left) and EMT (right) models respectively at *T*_s_ = 300 K for *φ*_i_ = 0°,54.74° and 90°. ELDs are decomposed into the T, HB, S contributions (see text). The collision energy is *E*_c_ = 2.76 eV and *θ*_i_ = 45°.

We next turn to the predicted ELDs for *T*_s_ = 300 K; their broad, structureless appearance results from the dominant influence of the random force, which is strongly temperature dependent.^8^ This broadening effect makes the ELDs predicted by the two models difficult to distinguish from one another. Despite this, we can use the T/HB/S categorisation scheme as before. The decomposition of the total specular ELDs at 300 K into the three contribution is shown in Fig. 9. As for the simulations at *T* = 70 K, the contribution of subsurface scattering events is stronger when *φ*_i_ = 0° but remains unresolved due to the broadening effect of the random force. The full-dimensional nature of the EMT-PES explicitly describes thermal displacement of the W lattice. This makes the S-scattering predicted by the EMT-PES method at *T*_s_ = 300 K less sensitive to *φ*_i_ compared to those predicted by the CRP-PES model. For the EMT-PES method, the contribution of S-scattering decreases from 28% to 18% when the surface temperature increases from 70 K to 300 K. By contrast, the CRP-PES approach predicts 32% S-scattering independent of surface temperature – see Table 1.

Both approaches provide similar mean energy losses, although it has to be noticed that both models differ in many aspects: (i) the DFT data and PES construction method (ii) the electron density (iii) the coupling to surface phonons. Despite these differences, they lead to a very good agreement for ELDs at 300 K and to qualitative agreement at 70 K. Further work, which would need support by experiments, is obviously necessary to evaluate the impact of each models assumptions. Nevertheless, the similarity of the information they give on the H/W(110) scattering dynamics is striking.

## 5 Conclusion

Molecular dynamics simulations with electronic friction treated at the level of LDFA have been implemented to investigate the dynamics of H scattering from W(110). Two different DFT based models have been used to describe both the adiabatic interactions as well as the frictional forces used to describe ehp excitation. Our findings show that the reduced dimensionality of the CRP-PES approach causes the energy loss distribution to be more sensitive to the initial azimuthal angle. However, since the random force has such a strong influence on the appearance of the ELDs at elevated temperatures, the frozen lattice approximation used in the GLO of the CRP-PES approach does not introduce large errors. In fact, it is only possible to distinguish the predictions of the two models at low temperature. The higher background electron density of the EMT-PES model shifts the ELDs to slightly higher energy losses. This also results in a higher sticking probability along with a smaller contribution of subsurface scattering to the signal. The mean energy losses predicted by the two models are similar.

Despite their very different assumptions, both models provide similar scattering dynamics and predict a subsurface scattering channel for specular H atoms. The simulations predict that the optimum conditions to detect subsurface scattering is to cool the surface with liquid nitrogen or helium and employ projectiles travelling along the [001] direction.

## Conflicts of interest

There are no conflicts to declare.

## Supplementary Material

CP-024-D2CP01850K-s001

CP-024-D2CP01850K-s002

CP-024-D2CP01850K-s003

CP-024-D2CP01850K-s004
