# Supplementary figures and images for: H atom scattering from W(110): A benchmark for molecular dynamics with electronic friction
Source: Phys Chem Chem Phys. 2022 Aug 25;24(35):20813–9. doi: 10.1039/d2cp01850k (PMC9472596; doi:10.1039/d2cp01850k)

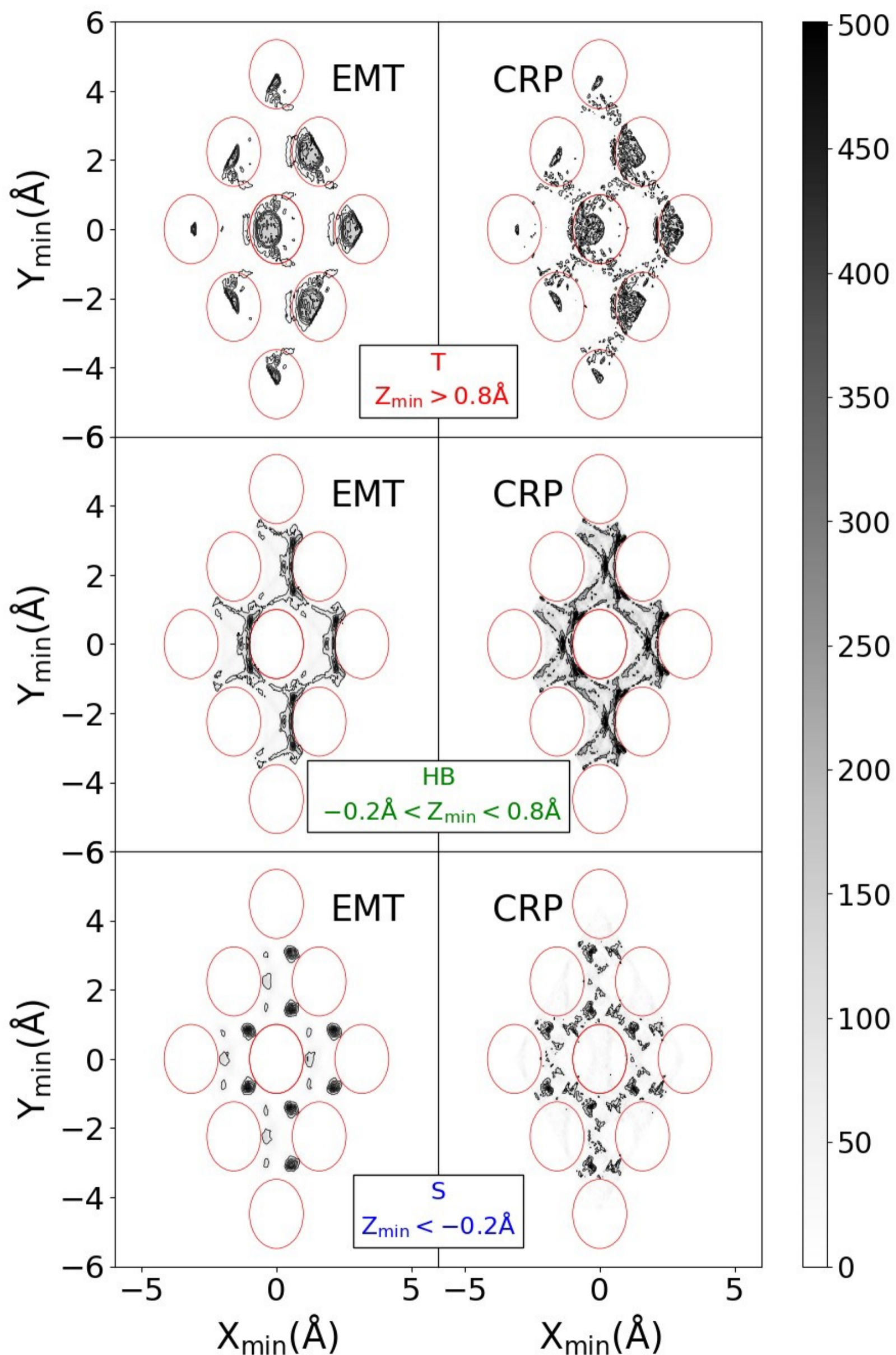

Supplement: CP-024-D2CP01850K-s002 [file CP-024-D2CP01850K-s002.pdf]

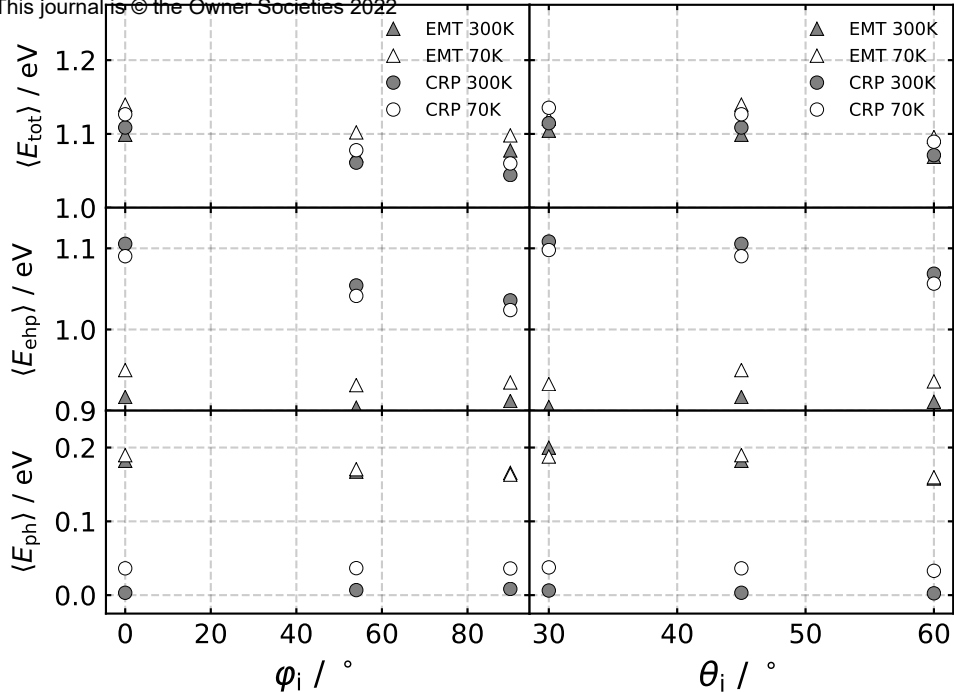

Supplement: CP-024-D2CP01850K-s003 [file CP-024-D2CP01850K-s003.pdf]
