# Supplementary material for: H atom scattering from W(110): A benchmark for molecular dynamics with electronic friction
Source: Phys Chem Chem Phys. 2022 Aug 25;24(35):20813–9. doi: 10.1039/d2cp01850k (PMC9472596; doi:10.1039/d2cp01850k)
Supplement: CP-024-D2CP01850K-s004 [file CP-024-D2CP01850K-s004.pdf]

Electronic Supplementary Material (ESI) for Physical Chemistry Chemical Physics.
This journal is © the Owner Societies 2022

Supplementary Information

H atom scattering from W(110): A benchmark for molecular dynamics with electronic friction.

Raidel Martin-Barrios[a,b], Nils Hertl[c,d], Oihana Galparsoro[e], Alexander Kandratsenka[c,d], Alec M. Wodtke[c,d], and Pascal Larrégaray[a]

[a]Univ. Bordeaux, CNRS, Bordeaux INP, ISM, UMR5255, F-33400, France.
[b]Dynamical processes in Atomic and Molecular Systems (DynAMoS), Facultad de Física, Universidad de la Habana, La Habana 10400, Cuba.
[c]Max-Planck-Institut für Multidisziplinäre Naturwissenschaften, Am Faßberg 11, Göttingen, Germany.
[d]Institut für Physikalische Chemie, Georg-August-Universität, Tammannstraße 6, Göttingen, Germany.
[e]Polimero eta Material Aurreratuak: Fisika, Kimika eta Teknologia Saila, Kimika Fakultatea, Euskal Herriko Unibertsitatea (UPV/EHU) Lardizabal Pasealekua 3, 20018 Donostia-San Sebastián, Spain

Figure S1 displays one-dimensional cuts of the EMT-PES and the CRP-PES along with the input data which have been used to optimise the EMT energy expressions for an optimised W(110) slab at 0 K. Under those conditions, the interpolated CRP-PES provides a better accuracy compared to the EMT-PES. This higher accuracy comes at the cost of a reduced dimensionality which prevents explicit treatment of lattice motion—see main text for details.

Figure S2 displays, for the H/W(110) system, the distributions of (X,Y) coordinates on the surface at the minimum turning point, for the T- ($Z_{min} > 0.8$ Å), HB- (-0.2 Å$> Z_{min} > 0.8$ Å) and S- ($Z_{min} < -0.2$ Å) contributions . For the T-component the minimum rebound occurs on top of a tungsten atom while it spans the Hollow and Bridge areas for the HB component. For the S-component, the rebound also appears in the Hollow and Bridge areas, but, in this case, between the surface and on the second layer of tungsten atoms. The results are in good agreement for the EMT and CRP models.

In Figure S3, the mean energy loss of all scattering events is split into the mean energy that is transferred by the H atom to the electrons and to the phonons. Note that while the total mean energy loss is in good agreement between both models, the individual parts show more pronounced differences between each other. The energy transfer to the lattice is negligibly small in the CRP model and also depends on the surface temperature. In the full-dimensional EMT-based simulation the mean energy transfer to the lattice is approximately 1/6 of the entire mean energy loss and can be reasonably rationalised with a binary collision model. [1]

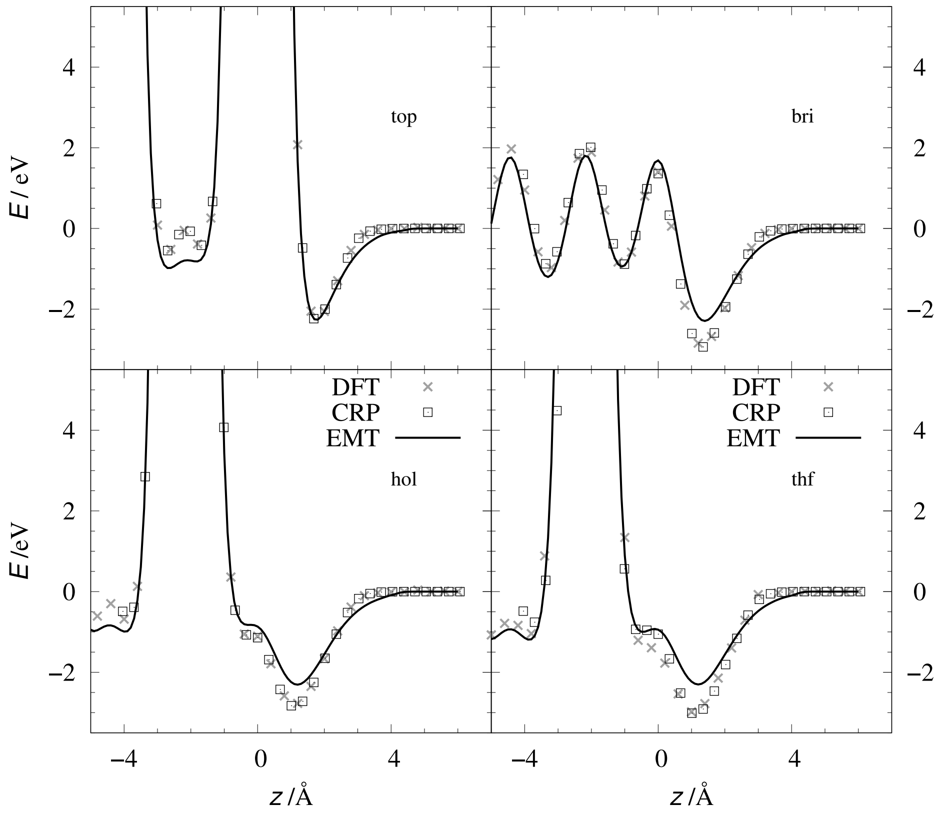

Figure S1: Potential energy plotted against the z coordinate of the H atom at the high symmetry sites, depicted in Figure 1 in the main text. The grey crosses mark the DFT data used for the construction of the EMT-PES, which is represented as solid, black line. The open squares represent the CRP-PES.

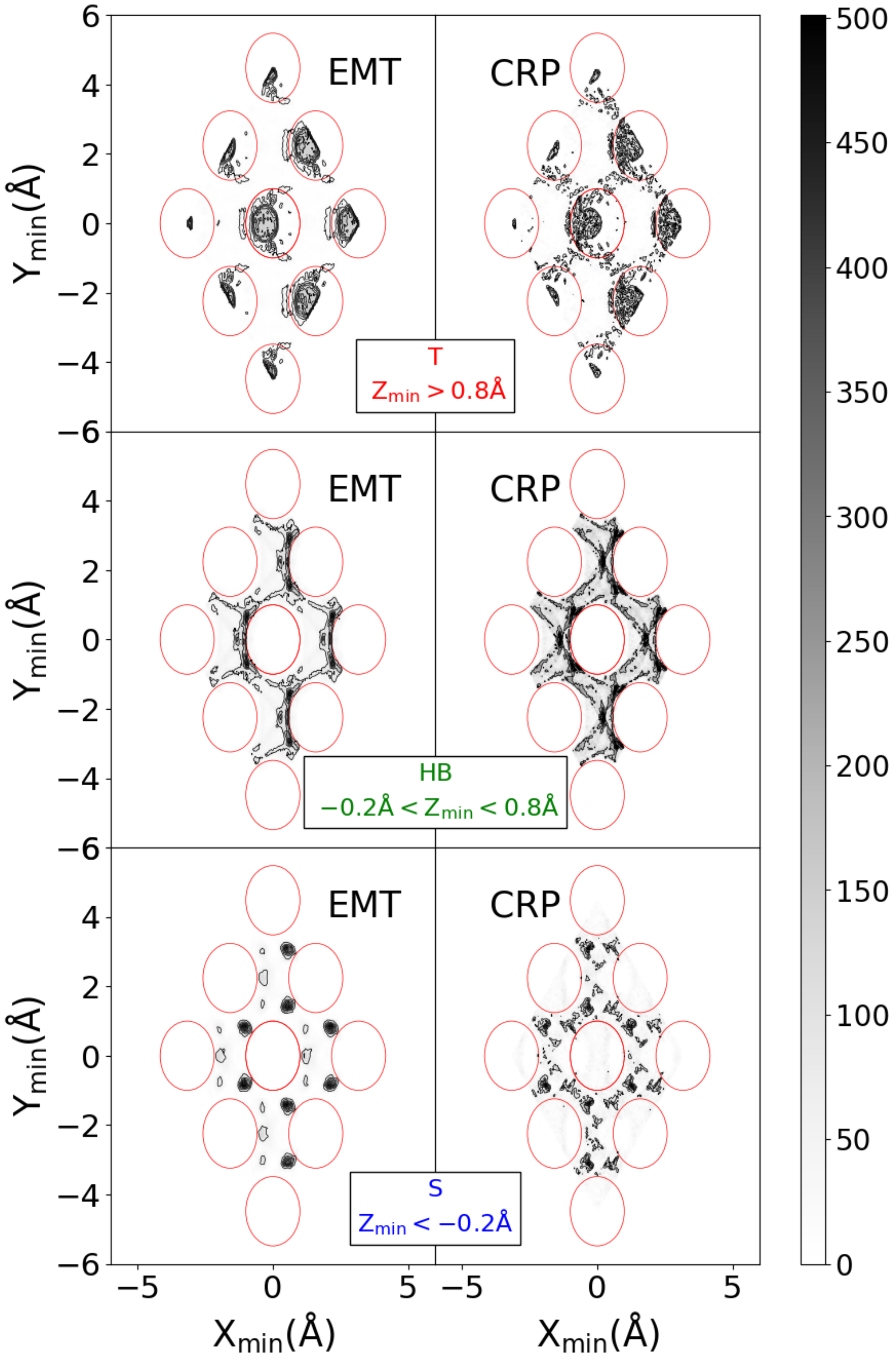

Figure S2: Distributions of (X,Y) coordinates of the minimun turning points, for the T-, HB- and S-contributions for Effective Medium Theory (EMT, left) and Corrugation Reduction Procedure (CRP, right) models. The initial conditions for the simulations are $E_c = 2.76$ eV, $\varphi_i = 0°$, $\theta_i = 45°$, $T_s = 70\,$K. The red circles represent the tungsten surface atoms at their equilibrium positions. The black contour lines are separated every 50 units and the areas with a value greater than 500 are excluded.

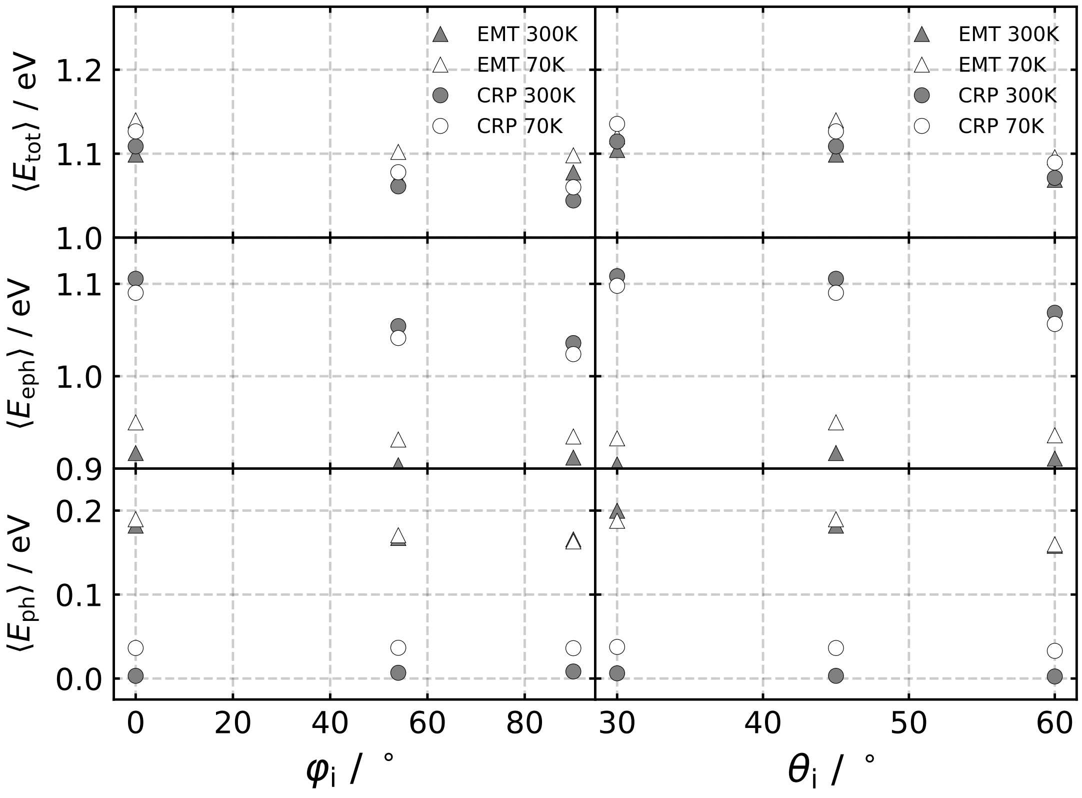

Figure S3: Decomposition of the mean energy loss $\langle E_{\mathrm{tot}} \rangle$, depicted in the first row, into the two possible energy transfer channels, i.e. ehp and phonon excitation for all investigated φ_{i} and θ_{i}. The second and third row show the mean energy loss due to ehp excitation and phonon excitation, respectively.

References

[1] E. K. Grimmelmann, J. C. Tully and M. J. Cardillo, *The Journal of Chemical Physics*, 1980, **72**, 1039–1043.
